# Elucidating the Formation and Structural Evolution of Platinum Single-Site Catalysts for the Hydrogen Evolution Reaction

**DOI:** 10.1021/acscatal.1c05958

**Published:** 2022-02-23

**Authors:** Peng Tang, Hyeon Jeong Lee, Kevin Hurlbutt, Po-Yuan Huang, Sudarshan Narayanan, Chenbo Wang, Diego Gianolio, Rosa Arrigo, Jun Chen, Jamie H. Warner, Mauro Pasta

**Affiliations:** † Department of Materials, University of Oxford, Parks Road, Oxford OX1 3PH, United Kingdom; ‡ Oxford Suzhou Centre for Advanced Research, 388 Ruoshui Road, Suzhou 215123, Jiangsu Province, P. R. China; § Diamond Light Source Limited, Harwell Science and Innovation Campus, Didcot, Oxfordshire OX11 0DE, United Kingdom; ∥ School of Science, Engineering and Environment, University of Salford, Manchester M5 4WT, United Kingdom; ⊥ Materials Graduate Program, Texas Materials Institute, The University of Texas at Austin, 204 East Dean Keeton Street, Austin, Texas 78712, United States; # Walker Department of Mechanical Engineering, The University of Texas at Austin, 204 East Dean Keeton Street, Austin, Texas 78712, United States

**Keywords:** hydrogen evolution reaction, platinum, single-site catalysts, agglomerates, operando X-ray absorption spectroscopy

## Abstract

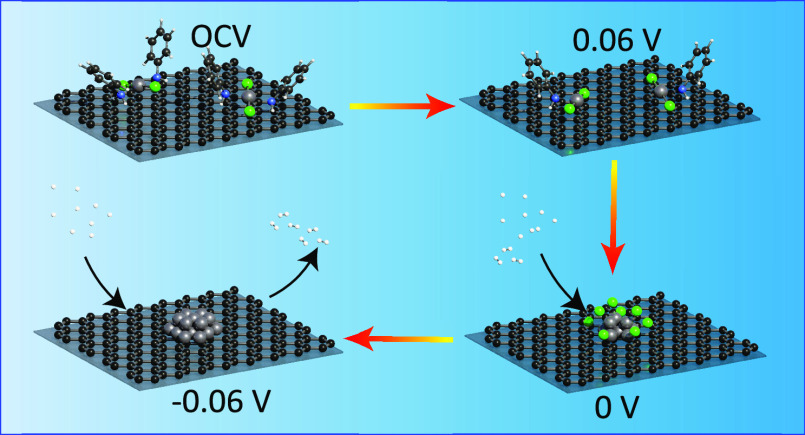

Platinum single-site catalysts (SSCs) are a promising technology for the production of hydrogen from clean energy sources. They have high activity and maximal platinum-atom utilization. However, the bonding environment of platinum during operation is poorly understood. In this work, we present a mechanistic study of platinum SSCs using operando, synchrotron-X-ray absorption spectroscopy. We synthesize an atomically dispersed platinum complex with aniline and chloride ligands onto graphene and characterize it with ex-situ electron microscopy, X-ray diffractometry, X-ray photoelectron spectroscopy, X-ray absorption near-edge structure spectroscopy (XANES), and extended X-ray absorption fine structure spectroscopy (EXAFS). Then, by operando EXAFS and XANES, we show that as a negatively biased potential is applied, the Pt–N bonds break first followed by the Pt–Cl bonds. The platinum is reduced from platinum(II) to metallic platinum(0) by the onset of the hydrogen-evolution reaction at 0 V. Furthermore, we observe an increase in Pt–Pt bonding, indicating the formation of platinum agglomerates. Together, these results indicate that while aniline is used to prepare platinum SSCs, the single-site complexes are decomposed and platinum agglomerates at operating potentials. This work is an important contribution to the understanding of the evolution of bonding environment in SSCs and provides some molecular insights into how platinum agglomeration causes the deactivation of SSCs over time.

## Introduction

Catalysts for the electrochemical hydrogen evolution reaction (HER) are desirable to produce hydrogen from clean sources like solar energy.^1,2^ Platinum is among the most active HER catalysts known in acidic media.^3^ Platinum is a heterogeneous catalyst with the HER occurring only on the metal surface.^4^ This can be prohibitively inefficient because platinum is so rare and expensive.^5^

Single-site catalysts (SSCs) are an emerging type of heterogeneous catalysts in which isolated metal sites are supported on suitable substrates.^6,7^ Each metal site is well separated from other metal atoms by a distance greater than the length of the metallic chemical bond.^8,9^ Platinum SSCs can be prepared by synthesizing a platinum complex and adsorbing the whole complex to a suitable substrate.^10−12^ They have extremely high atom utilization because every catalytic atom is exposed to the reactants and available for reaction.^13,14^ We choose to distinguish these structures from single-atom catalysts (SACs), which have also been shown to promote a broad range of catalytic reactions,^15−17^ since the Pt SSCs we investigate are complexes of several atoms surrounding the catalytically active metal atom.^18,19^

Despite the promise of platinum SSCs for the HER, the bonding environment of platinum in these devices before and during operation is poorly understood.^20−23^ Establishing the structure–property relationship between the platinum bonding and the electrochemical performance of the catalyst is critically important to enabling design of tailored, high-performing platinum SSCs.^24,25^ Furthermore, platinum SSCs for the HER have poor long-term stability, but the cause of their degradation is poorly understood.^26^ Operando spectroscopy methods are a power tool to determine at the molecular level why these catalysts deactivate over time.^27−30^

In this work, we examine the model SSC system of a platinum complex with aniline and chloride ligands (*trans*-dianilinedichloroplatinum(II)) adsorbed onto graphene. Graphene is an excellent substrate because of its high electronic conductivity, chemical stability^31^ and the good contrast it provides with platinum in electron microscopy.^32^ At the same time, aniline is stabilized via adsorption onto graphene through π–π stacking interactions with the molecule’s aromatic ring.^10^ Furthermore, aniline readily complexes with platinum, and the molecule’s large size provides a steric hindrance that can prevent platinum agglomeration.^33,34^

We characterize the physical structure of the as-synthesized SSC with ex-situ scanning transmission electron microscopy (STEM) and X-ray diffractometry (XRD). We examine the SSC’s oxidation state and bonding environment with X-ray photoelectron spectroscopy (XPS), X-ray absorption near-edge structure spectroscopy (XANES), and extended X-ray absorption fine structure spectroscopy (EXAFS). Finally, we use chronoamperometry with operando EXAFS and XANES, fitted with structures calculated using density-functional theory (DFT), to study how platinum’s bonding environment and coordination number change as a function of applied potential during the HER. This work is a good demonstration of using operando X-ray spectroscopy techniques to probe the atomic-level structural evolution of platinum SSCs for the HER during operation. This is an important contribution to the understanding of the synthesis and stability of platinum SSCs.

## Results and Discussion

### Synthesis and Physicochemical Characterization

**Figure 1:**
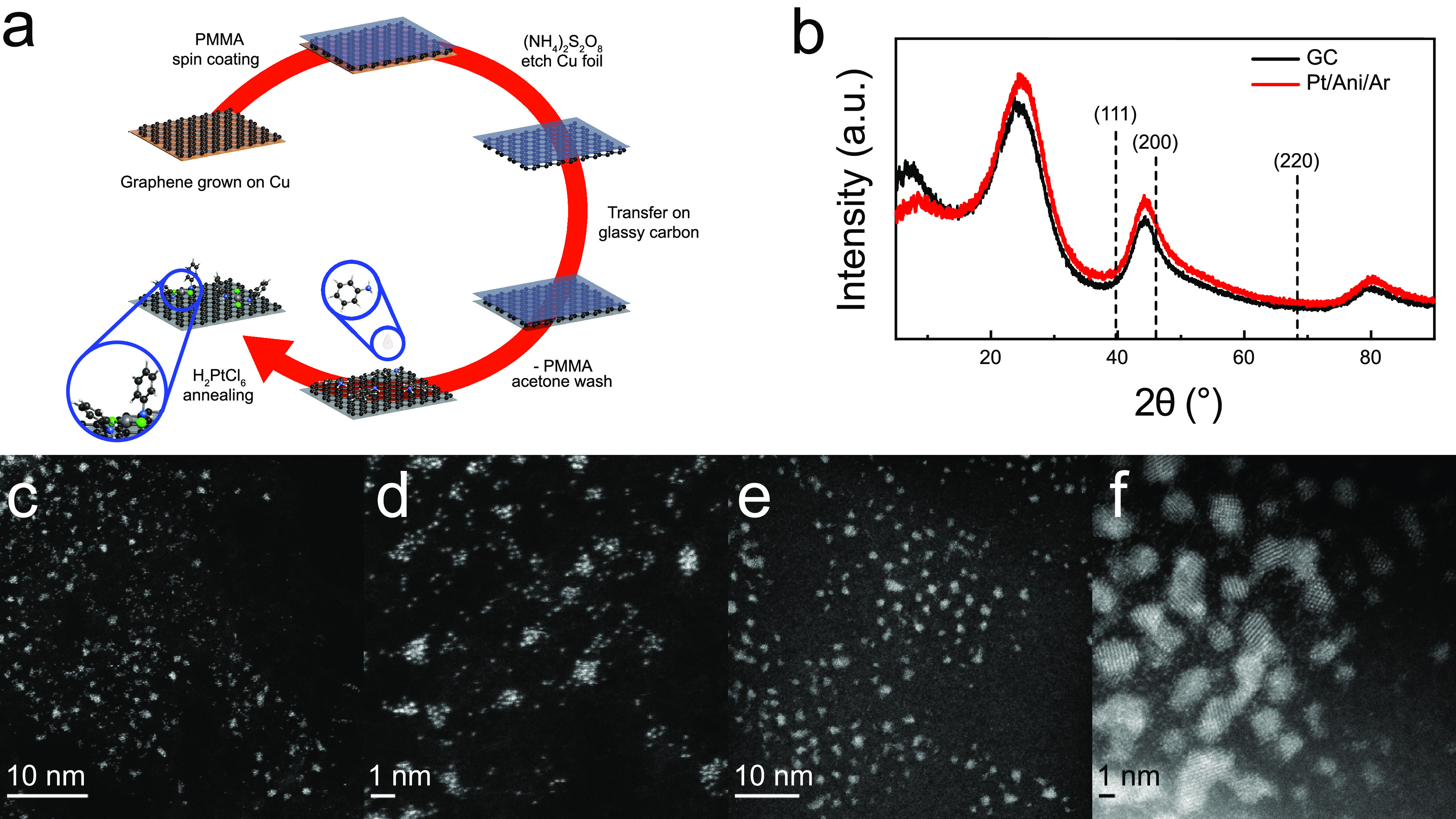
(a) Schematic illustration of the synthesis process (5 steps) for Pt SSC (gray, Pt; green, Cl; blue, N; black, carbon; white, H). Step 1: graphene nanosheets are grown by chemical vapor deposition (CVD) on copper. Step 2: copper foil is etched after spin coating with poly(methyl methacrylate) (PMMA) on top. Step 3: Thin-layer graphene coated with PMMA is then transferred onto a polished glassy carbon (GC) electrode. Step 4: after washing the PMMA with acetone and heating to 250 °C to remove trace organic contaminants, aniline is drop cast onto the graphene-coated GC electrode and dried. Step 5: chloroplatinic acid in ethanol is then drop cast and annealed under an argon atmosphere. This sample is referred to as the (Pt/Ani/Ar) sample. Control sample (Pt/Ar) is prepared by the same method but without aniline. (b) X-ray diffraction patterns for pristine GC and a (Pt/Ani/Ar) sample on GC; low-magnification to high-magnification atomic resolution HAADF-STEM images of (Pt/Ani/Ar) (c, d) and (Pt/Ar) (e, f).

The platinum SSC is prepared according to a modified impregnation method described in detail in the Experimental Procedures section in the Supporting Information and outlined in Figure 1a.

High-angle annular dark-field scanning transmission electron microscopy (HAADF-STEM) of (Pt/Ani/Ar) and (Pt/Ar) are shown in Figure 1c and 1d and 1e and 1f, respectively. (Pt/Ani/Ar) contains atomically dispersed platinum with an average areal number density of about 0.5 nm^–2^ (Supplementary Figures S1 and S2). Some aggregates of platinum are present;^34^ these are generally of size 1 nm or smaller (Supplementary Figure S2a). (Pt/Ar) mainly contains larger aggregates and nanoparticles that are up to about 4 nm across (Supplementary Figure S2b).

The XRD patterns for the glassy carbon (GC) electrode and for (Pt/Ani/Ar) are shown in Figure 1b.^35^ The two patterns match well. In particular, the pattern for (Pt/Ani/Ar) lacks the intense peaks at about 39.8°, 46.2°, and 68.5° expected for the (111), (200), and (220) reflections in crystalline platinum (PDF# 04-0802). This implies that the HAADF-STEM images, which show no platinum nanoparticles in (Pt/Ani/Ar), are representative of the bulk sample. The STEM images show that treatment with aniline promotes the formation of more atomically dispersed platinum.

### Ex-Situ XPS and XAS

**Figure 2:**
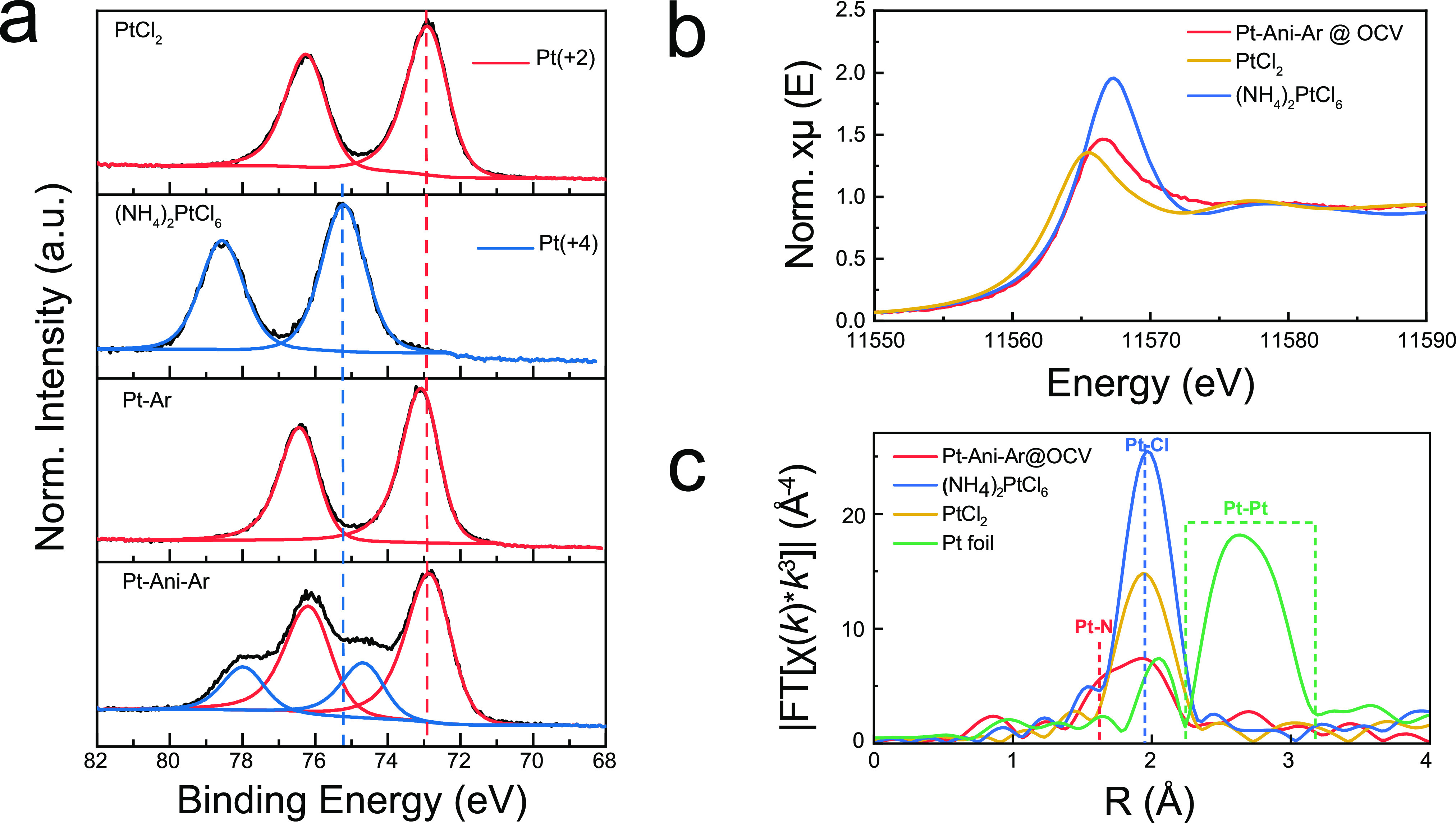
XPS spectra of Pt 4f (a) and XANES spectra (b) recorded at the Pt L_3_ edge for the synthesized Pt SSC and control sample (Pt/Ar) compared with reference standards in oxidation states of platinum(II) and platinum(IV). Vertical dashed red and blue lines in a indicate the peak positions of Pt_7/2_ at the oxidation state of platinum(II) and platinum(IV). (c) Corresponding *k*^3^-weighted Fourier-transformed Pt L_3_-edge EXAFS spectra of samples in b and standard Pt foil. Vertical dashed lines or area show the positions of Pt–N (red), Pt–Cl (blue), and Pt–Pt (green) peaks.

The platinum oxidation state was investigated by XPS, and the spectra for (Pt/Ani/Ar) and (Pt/Ar) are shown in Figure 2a. Two standards are also shown: ammonium hexachloroplatinate(IV) ((NH_4_)_2_PtCl_6_) and platinum(II) chloride (PtCl_2_). The platinum 4f_7/2_ peak in PtCl_2_ has a binding energy of 72.9 eV, consistent with the +2 oxidation state. The (NH_4_)_2_PtCl_6_ has a higher binding energy of 75.2 eV, which is also expected for its high oxidation state of +4. The spectrum for (Pt/Ani/Ar) is well fitted by a 74% contribution from a peak centered at 72.8 ± 0.1 eV and a 26% contribution from a peak centered at 74.6 ± 0.1 eV (Figure 2a and Supplementary Figure S5b). These contributions likely arise from platinum(II) and platinum(IV),^36^ respectively, indicating that the sample is a mixture of the two oxidation states.^24,25,37^ (Pt/Ar) is well fitted by a single peak centered at 73.0 ± 0.1 eV arising from the +2 oxidation state (Supplementary Figure S6).^38,39^ These oxidation-state assignments are consistent with the XANES spectra shown in Figure 2b. Here, reference spectra for PtCl_2_ (+2) and (NH_4_)_2_PtCl_6_ (+4) have white line peak positions at 11 565.8 and 11 567.7 eV, respectively. The spectrum for (Pt/Ani/Ar) has a single peak centered at 11 566.7 eV. This is expected of a mixture of platinum(II) and platinum(IV) and is consistent with the XPS data.^37,38^

The EXAFS spectra plotted in Figure 2c (along with those of the standards) give information about the platinum bonding environment. The standard samples of PtCl_2_ and (NH_4_)_2_PtCl_6_ have one dominant peak in the range from 1.5 to 2.3 Å with the center at 1.96 Å, suggesting that this peak can be associated with Pt–Cl bonding. The most intense peak in the spectrum for (Pt/Ani/Ar) is at 1.96 Å with a shoulder at 1.66 Å. The peak at 1.96 Å should arise from the presence of Pt–Cl bonds. This shoulder peak at 1.66 Å is indicative of platinum–nitrogen bonding.^40,41^ Finally, there is the absence of obvious Pt–Pt bonding at 2.73 Å (green curve in Figure 2c), which also suggests that the platinum atoms are mainly isolated from each other. We therefore interpret these data to mean that the aniline and chloride coordinate to the platinum to form a complex molecule (Supplementary Figure S7).

These XPS and XANES data indicate that (Pt/Ani/Ar) and (Pt/Ar) are reduced in situ from platinum(IV) (the oxidation state of platinum in the precursor H_2_PtCl_6_) to platinum(II). The cause of the reduction of Pt(IV) to Pt(II) is the ethanol used as a solvent to prepare the H_2_PtCl_6_ solution (Supplementary Figure S8).^42^ However, in (Pt/Ani/Ar), the platinum forms a complex with aniline (as confirmed by the bonding data from the EXAFS spectra). This is the reason that (Pt/Ani/Ar) is only partially reduced with platinum(IV) still present. The STEM, XPS, XANES, and EXAFS data suggest that the aniline-treated sample contains atomically dispersed platinum in the form of molecular complexes (Pt single-site complexes) physisorbed onto the graphene substrate.

### Operando XAS Measurements

**Figure 3:**
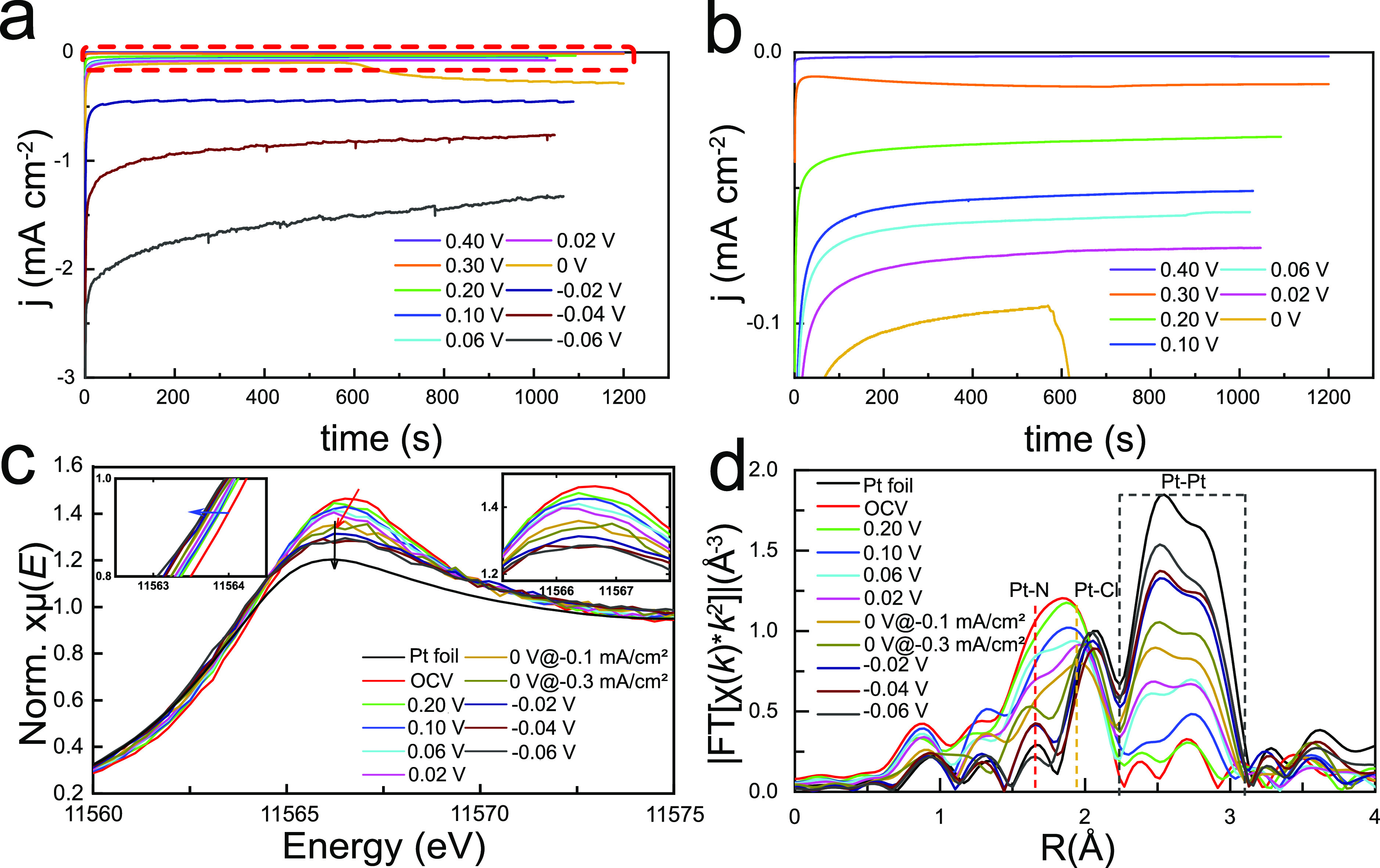
(a) Stable chronoamperometric curves of Pt single-site complex at various applied potentials. (b) Enlarged red dashed area in a shows the chronoamperometric curves at a current density smaller than 0.1 mA cm^–2^. (c) Operando XANES spectra recorded at the Pt L_3_ edge of the synthesized Pt single-site complex at different working potentials for the HER in 0.5 M H_2_SO_4_. (Insets) Energy shifts of the Pt L_3_ absorption edge (left) and white-line peak (right). (d) Corresponding *k*^2^-weighted Fourier-transformed EXAFS spectra (magnitude component). Vertical dashed lines or area show the Pt–N (red), Pt–Cl (orange), and Pt–Pt (gray) peaks.

Operando hard XAS at the platinum L_3_ edge was performed at different potentials to investigate the evolution of the platinum single-site complex’s oxidation state and chemical bonding environment during the HER.^23,30,43^ A customized three-electrode cell setup (Supplementary Figure S10) was mounted to investigate the aniline-treated sample (Pt/Ani/Ar). The chronoamperometric performance of (Pt/Ani/Ar) at various potentials is shown in Figure 3a (with an enlarged view shown in Figure 3b). The areal current densities are shown for at least 1000 s of operation at each of 10 voltages: 0.4, 0.3, 0.2, 0.1, 0.06, 0.02, 0, −0.02, −0.04, and −0.06 V versus the reversible hydrogen electrode (RHE). The open-circuit voltage (OCV) is 0.6 V versus RHE. None of the tests at a positive potential have an areal current density greater than 0.1 mA cm^–2^. This is to be expected since the HER is only thermodynamically favorable below 0 V versus RHE. Indeed, the data for the test at 0 V show a minimal areal current density until about 600 s (out of approximately 1200 s in total) when the current jumps suddenly and then gradually increases to about −0.3 mA cm^–2^. The areal current densities for the subsequent potentials of −0.02, −0.04, and −0.06 V versus RHE are −0.5, −0.9, and −1.5 mA cm^–2^, respectively. The Tafel slope, calculated using the 4 negative potentials, is 60 mV dec^–1^. This is comparable to the Tafel slope of platinum nanoparticles for the HER.^44,45^

The average of eight XANES spectra, collected during operation at each potential, is shown in Figure 3c. These data show that the oxidation state of platinum decreases from a mixture of +4 and +2 to 0. When the potential is decreased from 0.6 (OCV) to 0 V versus RHE, the positions of both the white line (red arrow and right inset in Figure 3c) and the absorption edge (left inset in Figure 3c) in the XANES spectra show a negative shift of 0.4 eV. Those shifts suggest a continuous decrease of platinum valence from a mixture of platinum(II) and platinum(IV) to metallic platinum.^23,46^

The bonding environment of the platinum also changes as the applied potential varies, as shown in the EXAFS spectra in Figure 3d. As the potential is decreased from OCV to 0, the peaks at 1.66 and 1.96 Å, arising from Pt–N and Pt–Cl bonds, respectively, both decrease in intensity. However, the peak arising from Pt–N (blue vertical line) decreases faster than that arising from Pt–Cl (orange vertical line). This indicates that as the potential is decreased from OCV and while the oxidation state of the platinum falls to 0, the aniline molecules are dissociating from the complex. The chloride ions are also dissociating from the platinum atoms but to a lesser extent compared to the aniline molecules. At 0 V, the SSC has two distinct EXAFS spectra (yellow and olive curves in Figure 3d and isolated in Figure S12): one at a low areal current density (−0.1 mA cm^–2^) before the onset of the HER and one at a higher areal current density (−0.3 mA cm^–2^) after the onset of the HER. Before the onset of the HER, the bonding environment still shows appreciable Pt–Cl bonding and some Pt–N bonding. After 600 s and the beginning of hydrogen evolution, almost all Pt–N bonding character is lost and the Pt–Cl bonding feature decreases significantly. The EXAFS spectrum is instead dominated by Pt–Pt bonding. The rapid breaking of Pt–N and Pt–Cl bonds at 0 V may be caused by hydrogen molecules aggregated on the SSC. It has been experimentally shown that the diffusion and agglomeration of platinum atoms can be induced by exposure to a hydrogen atmosphere.^47−50^ Since more reduced platinum structures after 600 s can boost the evolution of hydrogen by lowering the Gibbs free energy of hydrogen adsorption in acidic electrolytes,^23,51^ the sudden increase in current density here (the yellowish line discussed in Figure 3a) can probably be justified accordingly.

### Structure–Electrochemistry Correlation

**Figure 4:**
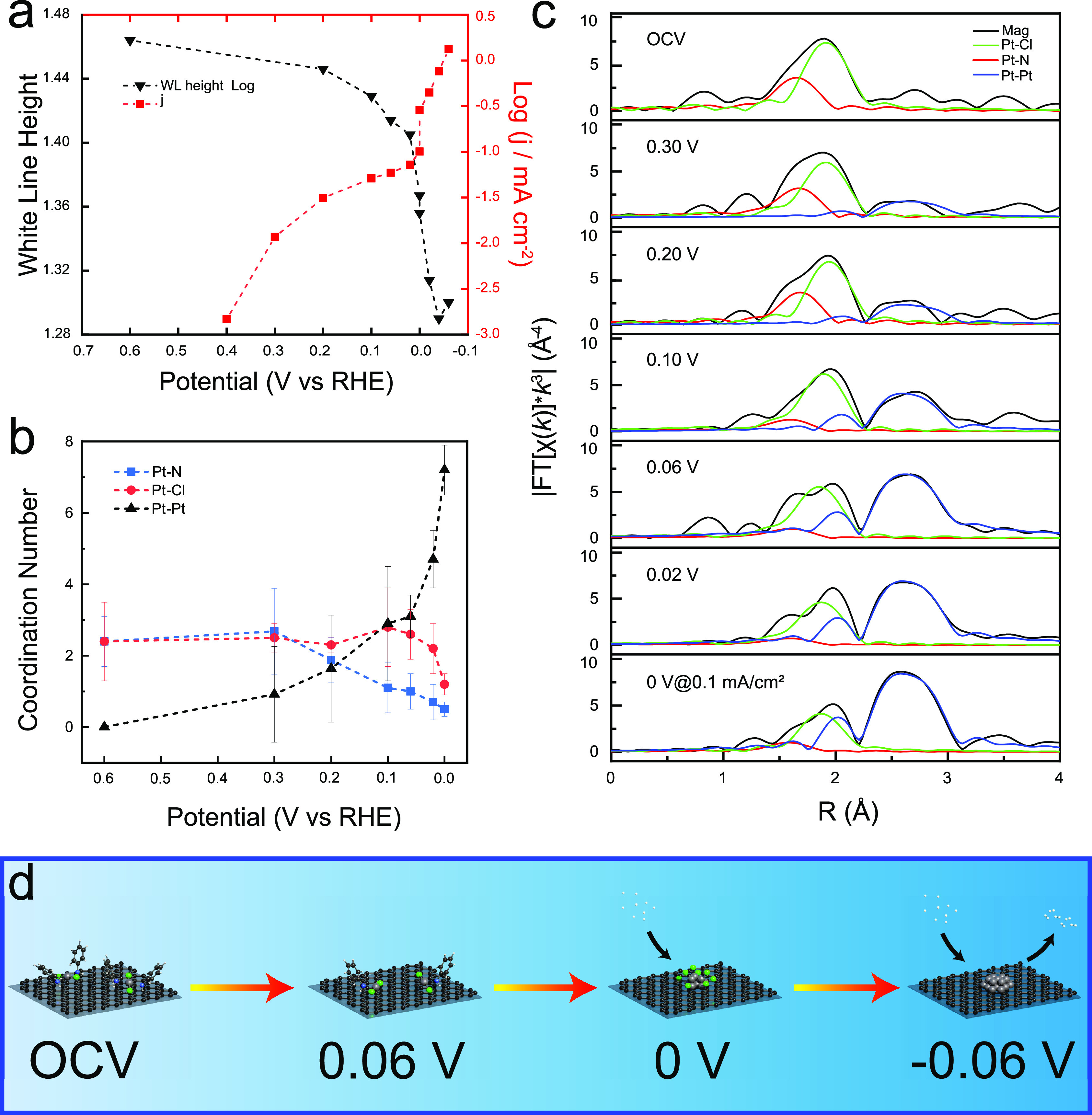
(a) Amplitude of the normalized white-line peak of the Pt L_3_-edge operando XANES spectra (black) and corresponding catalytic activity (red) at different potentials. Log(*j*) is calculated using a relatively stable current density at the end of each CA test. (b) Coordination number of Pt–N, Pt–Cl, and Pt–Pt fitting paths for operando EXAFS data of the Pt single-site complex at selected potentials by 0 V. Error bars in b represent the uncertainties of the fitting results. (c) Contribution of different paths (Pt–N, Pt–Cl, and Pt–Pt) to fit the corresponding EXAFS spectra. Details of the fitting results shown in *K*-space and *R*-space are found in Figure S13. (d) Proposed schematic model for the evolution of the (Pt/Ani/Ar) sample from a single-site complex to subnanoclusters based on the operando EXAFS analysis (gray, Pt; green, Cl; blue, N; black, carbon; white, H).

The previous section elucidated the evolution of the Pt single-site complex into metallic nanoclusters at potentials below 0 V; the details of this transformation need to be quantitatively examined together with the catalytic activity. The effect of applied potential on both the areal current density and the platinum oxidation state in the SSC is shown in Figure 4a. The height of the XANES normalized white lines is summarized (black plot in Figure 4a) to quantify the evolution of platinum’s oxidation state and the electron occupancy of platinum’s 5d orbitals.^52,53^ The white-line height decreases precipitously from OCV (0.60 V) to about 0 V versus RHE. This is consistent with the complete filling of the platinum 5d valence orbitals and the formation of metallic platinum.^23^ At the same potential, the corresponding areal current density increases precipitously as the HER becomes thermodynamically favorable, although they are still relatively low (from −0.001 to −0.08 mA cm^–2^). There is an obvious drop in white-line height at around 0 V, and the white-line height stabilizes at around 1.30 after 0 V, indicating the oxidation state of platinum remains as 0 at potentials below 0 V. Corresponding to the decrease in oxidation state, a significant increase in current density is also recorded at 0 V (red plot in Figure 4a).After the increase, a linear relationship between the base-10 logarithm of the areal current density and the potentials is presented.

EXAFS curve-fitting analysis on the platinum’s EXAFS spectrum at OCV allows for the determination of a plausible molecular structure for the platinum complex adsorbed onto graphene. Considering eight possible platinum complexes described by (C_6_H_5_NH_2_)_*x*_Cl_*y*_Pt for *x* = 1 or 2 and *y* = 1, 2, 3, or 4, the Pt–N and Pt–Cl bond lengths (about 2.05 and 2.30 Å, respectively) determined by density-functional theory modeling of the isolated molecular complexes are the starting point for the analysis. The operando EXAFS data are best described by the structural model for *trans*-dianilinedichloroplatinum(II), (C_6_H_5_NH_2_)_2_Cl_2_Pt(II), within the fitting range (*k*-range, 3–12.5 Å^–1^, *R*-range, 1.1–2.5 Å, misfit factor 0.8%). This structure is consistent with the XPS and XANES spectra of the (Pt/Ani/Ar) sample (most of the Pt is at an oxidation of +2) and is similar to the previously reported crystal (C_6_H_5_NH_2_)_2_PtCl_2_ complex prepared by mixing aniline with H_2_PtCl_6_.^54,55^

Performing EXAFS curve-fitting analysis (platinum’s first (1.5–2.1 Å) and second (2.3–3.1 Å) coordination shells) on the spectra collected at different potentials results in the curves in Figure 4b and Table S1, which show the average number of platinum, chlorine, and nitrogen atoms coordinated to each platinum atom (i.e., the coordination number) as a function of applied potential. Specifically, Figure 4c highlights the evolution of the EXAFS spectra while decreasing potential in the *R* space fitted with the contributions of the Pt–N, Pt–Cl, and Pt–Pt paths. At OCV, the coordination number for both chlorine and nitrogen is about 2.5. As the potential is lowered, the coordination number for nitrogen decreases to about 1.0 by a potential of 0.1 V versus RHE. It continues to decrease to about 0.5 by 0 V (but before the onset of the HER). The chlorine coordination number stays higher than the nitrogen coordination number. This indicates that the aniline molecules dissociate from the complex before the chloride ions do, likely due to the more ionic character and higher bond energy of the Pt–Cl bonds compared to the Pt–N bonds.^56^ The platinum coordination number increases as the potential is lowered, ultimately reaching a value of about 7.0 by 0 V versus RHE. This indicates the agglomeration of platinum as the potential is lowered (Supplementary Figure S14), which is also in line with the reduction of the oxidation state of platinum described in Figure 4a.^57^

Together these data elucidate how the platinum bonding environment in an SSC changes as a function of applied potential during operation, as described schematically in Figure 4d. As the potential is lowered from OCV, the dianilinedichloroplatinum(II) decomposes as the aniline dissociates from the platinum, followed by the dissociation of the chloride ions. Simultaneously, the 5d valence orbitals of platinum are filled. By the onset of the HER at 0 V versus RHE, the dianilinedichloroplatinum(II) complexes have completely dissociated and the metallic platinum(0) single atoms agglomerate into nanoclusters which catalyze the HER.

## Conclusions

In this work, we use operando, synchrotron X-ray absorption spectroscopy to characterize how the oxidation state and bonding environment of platinum changes in an SSC for the HER at the molecular level. First, we show successful synthesis of a platinum SSC, confirming the platinum is atomically dispersed by STEM and XRD. We observe a mixture of platinum(II) and platinum(IV) by XPS and XANES, and we confirm platinum coordination to aniline by ex-situ EXAFS. We then probe the oxidation state and molecular structure as a function of applied potential using operando EXAFS and XANES with synchrontron radiation. We find that as the potential is lowered from OCV the platinum oxidation state decreases, breaking bonds between platinum atoms and aniline ligands first. The Pt–Cl bonds also break but to a lesser extent than the Pt–N bonds. Finally, once the onset of the HER at 0 V versus RHE is reached, the platinum is completely reduced and metallic platinum atoms agglomerate to form catalytic nanoclusters. Our work suggests that many single-site catalysts that achieve atomically dispersed platinum by ligand coordination do not remain atomically dispersed during device operation. This demonstrates that researchers should be extremely careful when assuming that the bonding environment of the as-synthesized SSC will remain the same during device operation. Furthermore, this operando study establishes two important structure–property relationships for aniline-complex platinum SSCs. First, platinum exists as metallic platinum (that is, in the oxidation state 0) while catalyzing the HER. Second, the molecular structure of the platinum complexes is lost due to the reduction of the platinum. These findings highlight the necessity for operando techniques to follow the dynamic behavior of SSCs during operation and provide critical new insight into why SSCs deactivate over time. Such experiments can also provide an important contribution to an atomic understanding of the structural evolution of SSC systems as well as SACs for other critical electrochemical reactions, such as the oxygen reduction reaction and carbon dioxide reduction reaction. These results and the operando X-ray spectroscopy method used in this study will accelerate the development of stable, high-performance platinum SSCs for clean hydrogen production.

## Supporting Information Available

Experimental sections; additional STEM analysis and XPS data of (Pt/Ani/Ar) and (Pt/Ar); Raman spectra of (Pt/Ani/Ar); setup of the operando XAS experiment; details of EXAFS fitting results; STEM, XPS, and XAS of (Pt/Ani/Ar) sample post cycling (PDF)

## Notes

Source data are available from the corresponding author upon reasonable request.
