# Supplementary material for: Elucidating the Formation and Structural Evolution of Platinum Single-Site Catalysts for the Hydrogen Evolution Reaction
Source: ACS Catal. 2022 Feb 23;12(5):3173–80. doi: 10.1021/acscatal.1c05958 (PMC9086987; doi:10.1021/acscatal.1c05958)
Supplement: Supplementary file 1 — cs1c05958_si_001.pdf [file cs1c05958_si_001.pdf]

SUPPLEMENTARY INFORMATION

Elucidating the formation and structural evolution of platinum single-site catalysts for hydrogen evolution reaction

Peng Tang,[†] Hyeon Jeong Lee,[†] Kevin Hurlbutt,[†] Po-Yuan Huang,[†] Sudarshan Narayanan,[†] Chenbo Wang,[‡] Diego Gianolio,[¶] Rosa Arrigo,[§] Jun Chen,[†] Jamie H. Warner,[‖,⊥] and Mauro Pasta*,[†,‡]

†*Department of Materials, University of Oxford, Parks Road, Oxford OX1 3PH, United Kingdom*

‡*Oxford Suzhou Centre for Advanced Research, 388 Ruoshui Road, Suzhou 215123, Jiangsu Province, P. R. China*

¶*Diamond Light Source Ltd., Harwell Science and Innovation Campus, Chilton, Didcot, OX11 0DE, UK*

§*School of Science, Engineering and Environment, University of Salford, M5 4WT Manchester, U.K*

‖*Materials Graduate Program, Texas Materials Institute, The University of Texas at Austin, 204 East Dean Keeton Street, Austin, Texas, 78712, United States*

⊥*Walker Department of Mechanical Engineering, The University of Texas at Austin, 204 East Dean Keeton Street, Austin, Texas, 78712, United States*

E-mail: mauro.pasta@materials.ox.ac.uk

Experimental Section

Chemicals

Chloroplatinic acid hexahydrate (37.5% Pt basis), aniline (99.5%) and ammonium persulfate (98%) were purchased from Sigma-Aldrich. Ultra-pure water (18.2 MΩ cm^{-1}; by Direct-Q Water Purification System) was used for the electrochemical tests.

Synthesis of Pt single-atom/clusters at surface of graphene

The Pt single-site complex catalysts stabilised on the surface of thin-layer graphene by aniline molecules was prepared using a modified impregnation method.[1] Firstly, thin-layer graphene is grown by a CVD method, and transferred onto the surface of well-polished glassy carbon (GC).[2] Then, graphene surface is cleaned by annealing under Ar (70 sccm) / H_2 (5 sccm) flow at 250 ℃ for 2 hours. The remaining sulphur from ammonium persulfate and small organics are removed. Then samples with different Pt structures are synthesised on the clean surface of graphene. Sample one (**Pt/Ani/Ar**): 4 µL of aniline drop casted on a 1 cm^2 graphene nanosheets, dried in ambient conditions for 15 minutes, then drop casting 10 µL of 10 mM/L H_2PtCl_6 ethanol solution. It is then annealed in argon atmosphere at 100 °C for 1.5 h. Sample two (**Pt/Ar**): synthesised using the same procedure without drop casting aniline. For further comparison, samples are prepared by annealing (Pt/Ani/Ar) at 200 °C instead of 100 °C (**Pt/Ani/Ar/200 °C**), and replacing argon annealing condition with hydrogen annealing condition (**Pt/Ani/H_2**). After the annealing treatment, all samples are sequentially washed with 0.5 M sulfuric acid then deionized water, and dried under vacuum overnight.

Ex-situ STEM and XRD

Room-temperature annular dark-field scanning transmission electron microscopy was performed with a JEOL ARM200F at an accelerating voltage of 80 kV. Dwell times of 10-20

µs, a beam current of 35 pA, and a pixel size of 0.006 nm px^{-1} were used for imaging, with a convergence semi-angle of 25.5 mrad and collecting inner-outer angles 68 to 275 mrad. XRD patterns were collected using a monochromated Cu Kα X-ray source from 5° to 90° (2ϑ), at a scan rate of $5°min^{-1}$.

Ex-situ XPS and XAS

X-ray photoemission spectroscopy was conducted using a PHI VersaProbe III system generating monochromatic Al X-rays at 1486 eV. Survey scans and high-resolution elemental scans were acquired at pass energies of 224 eV and 55 eV respectively. The binding energies were calibrated using the C 1s line at 284.8 eV as reference. The XPS spectra were fit using Gaussian-Lorentzian and Voigt lineshapes (where asymmetry was pronounced), and quantified with the help of CasaXPS software. X-ray absorption fine structure (XAFS) measurements at the Pt L_3 edge were carried out at the B18 Core EXAFS beamline of Diamond Light Source. The Pt reference samples (300 mg) were finely ground, mixed homogeneously with five parts of cellulose and pressed into 13 mm diameter pellets. Commercial $(NH_4)_2PtCl_6$ (Sigma-Aldrich, 99.9%), $PtCl_2$(Sigma–Aldrich99.9 %) were used as references. All spectra were recorded in transmission mode at room temperature.

Electrochemical measurements

The catalytic activity of synthesised samples were evaluated under a three-electrode cell with graphite rod as counter electrode and Ag/AgCl (in 3M KCl) as reference electrode in 0.5M Ar/N_2-purged H_2SO_4 solution. A 0.2 mm thick well-polished glassy carbon plate (Sigma-Aldrich Company Ltd.) was firstly used to transfer and support the thin-layer graphene nanosheets. Then the GC supporting well-dispersed Pt catalysts with (1 cm × 1 cm) defined area was used as the working electrode. A metal clip was used to connect the working electrode with an external circuit. The chronoamperometric performances were recorded by a Ivium Technologies or Biologic VMP3 potentiostat. All potentials were calibrated versus

RHE using E (RHE) = E (Ag/AgCl) + 0.197 V + 0.0592×pH.

Operando XAS

EXAFS and XANES were performed at the B18 Core EXAFS beamline of Diamond Light Source. The measurements were carried out using the Cr-coated branch of collimating and focusing mirrors, and a Si(111) double-crystal monochromator. The size of the beam at the sample position was ca. 1mm (h)×1mm (v). Samples were measured both in static electrolyte and operando conditions. The data were collected in fluorescence mode, by means of a 36-element solid state germanium detector, the ion chamber at the front of the sample has been used for measurement of incoming photons (I_0 filled with a mixture of 80 mbar of Ar and 1020 mbar of He to optimise sensitivity at 15% efficiency). An electrochemical cell adapted to the B18 beamline of the UK's Synchrotron Diamond Light Source was designed for the operando study shown in Supplementary Figure S10. Before the operando electrochemical measurements, the Pt L_3 edge spectra were measured on contact with the liquid electrolyte. The operando XAFS spectra of the Pt L_3 edge (11564 eV) were obtained from 200 eV before the edge up to 750 eV after the edge (corresponding to 14 $Å^{-1}$ in k-space). The measuring time was 2 minutes per spectrum. When indicated, 8 repetitions at each potential were acquired and then merged to obtain a better signal to noise ratio. Data were normalised using the Athena[3] program with a linear pre-edge and polynomial post-edge background subtracted from the raw data. EXAFS fits were performed using ARTEMIS software.[3]

Computational methods

DFT calculations were performed using the Vienna ab initio Simulation Package (VASP).[4,5] We performed Γ-point only calculations using a plane-wave kinetic-energy cutoff of 520 eV with electronic and ionic convergence criteria of 10^{-5} eV and 0.05 eV $Å^{-1}$, respectively. The Perdew-Burke-Ernzerhof (PBE) functional was used for all structures.

Additional results and discussion

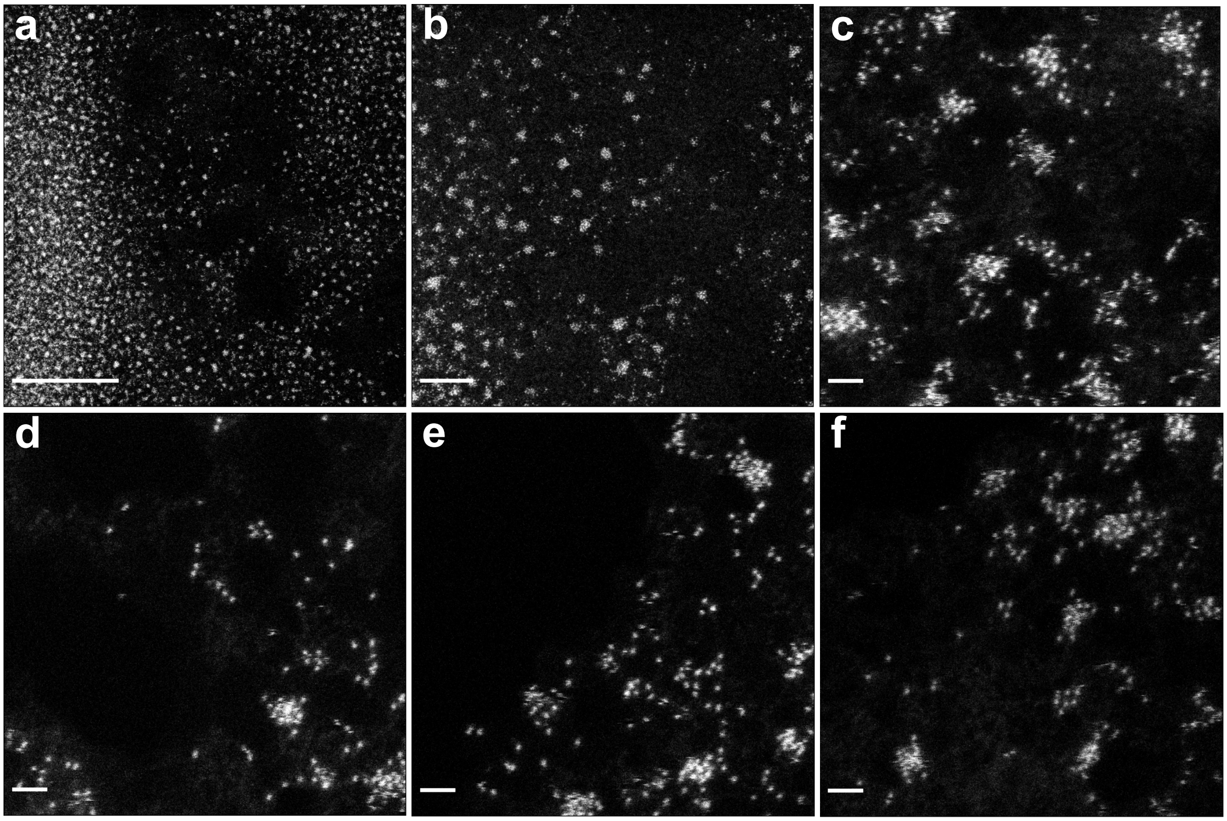

Figure S1. Low to high magnification atomic resolution HAADF-STEM images in different areas of the (Pt/Ani/Ar) sample to show the formation of single-site Pt is recorded widely; the scale bar is 20 nm (**a**), 5 nm (**b**) and 1 nm (**c**, **d**, **e**, **f**).

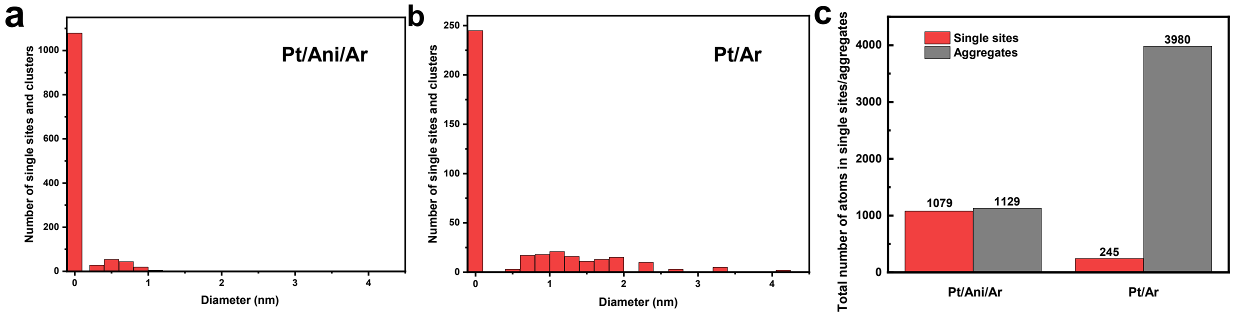

Figure S2. (**a**,**b**) Representative histograms quantifying the number of Pt single sites and aggregates diameter of (Pt/Ani/Ar) and (Pt/Ar) samples across an area of approximately 2000 nm^2 each from several STEM images.(**c**) Total number of Pt atoms in single-site Pt and Pt aggregates in (Pt/Ani/Ar) and (Pt/Ar). A Pt single site is defined as when the distance between the site and its nearest neighbour atoms is larger than a metallic Pt-Pt bond; this diameter is defined as 0 in the histograms.

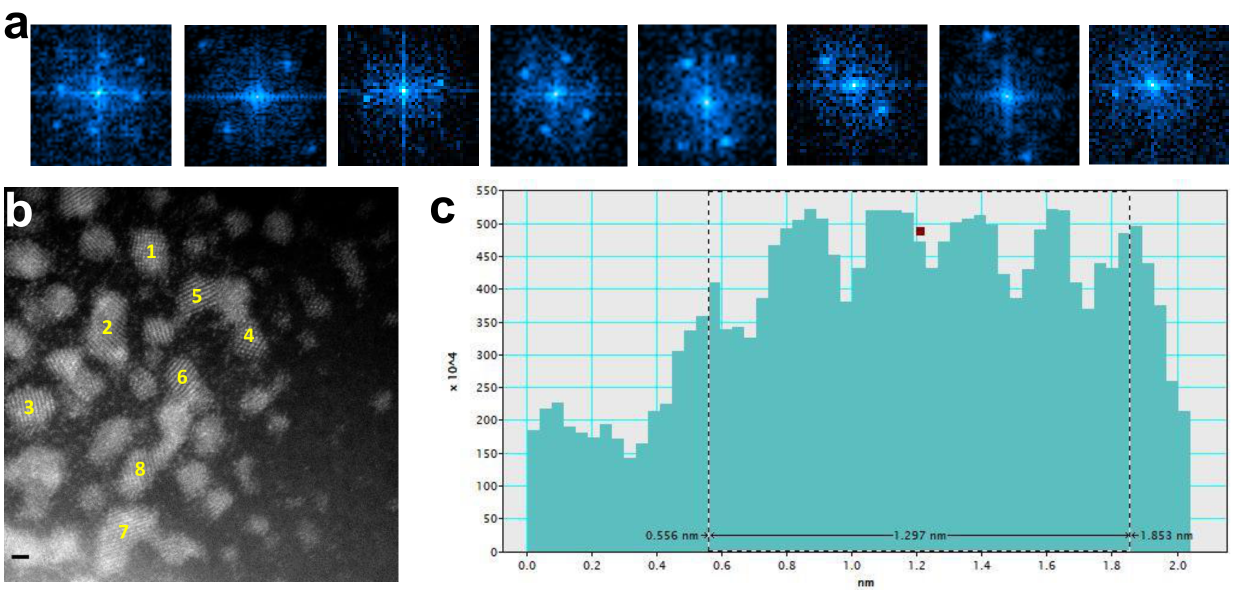

Figure S3. (**a**,**b**) Corresponding crystal orientations (**a**) of Pt nanoparticles in (Pt/Ar) samples labelled in its atomic STEM images in (**b**), which shows the crystal orientations in formed nanoparticles of (Pt/Ar) samples are random. (**c**) The lattice spacing of Pt {1,1,1} facet in STEM images is 0.25±0.01 nm measured by DigitalMicrograph software. It is a clear evidence to confirm the formed structures are Pt nanoparticles.

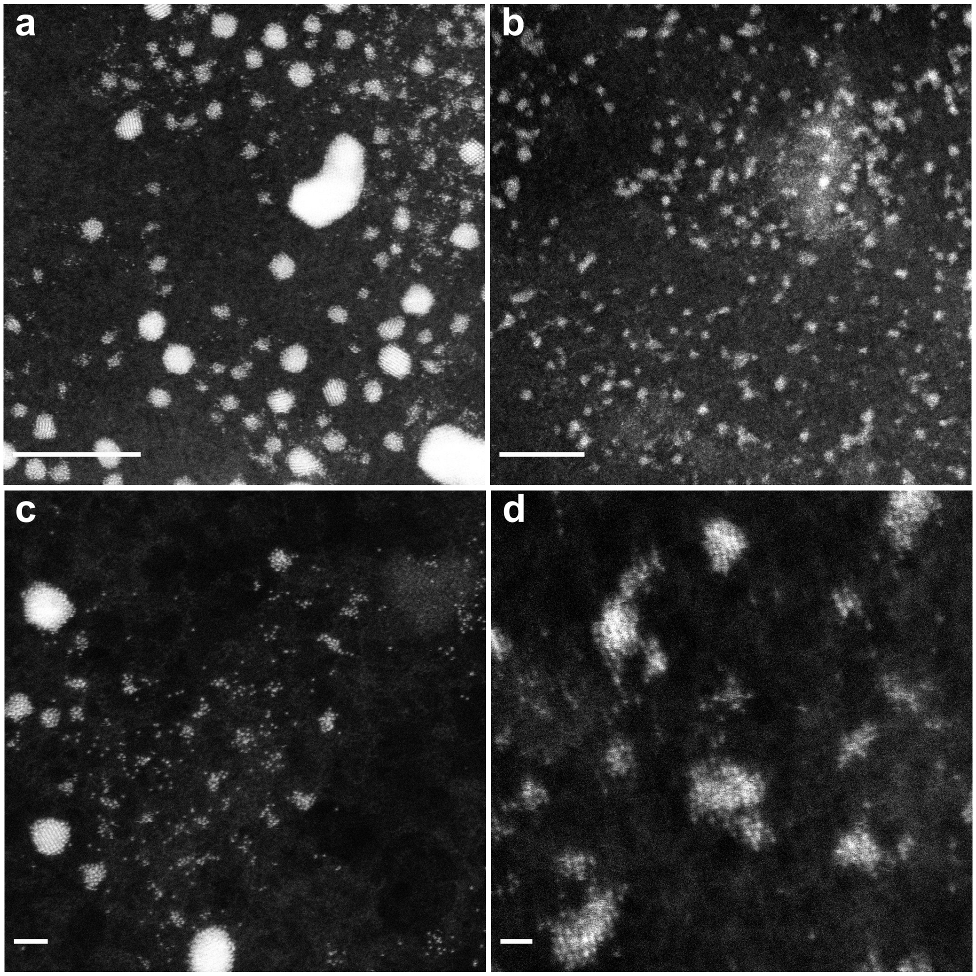

Figure S4. Low (top) to high (bottom) magnification atomic resolution HAADF-STEM images of the (Pt/Ani/H_2) sample (left two) and (Pt/Ani/Ar/200 °C) samples (right two); the scale bars are 10 nm (**a,b**), 2 nm (**c**) and 1 nm (**d**). The Pt/Ani/H_2 sample exhibited Pt nanoparticles 1-6 nm in size, although single atoms and clusters were also present. The formation of nanoparticles once the sample is annealed under hydrogen atmosphere implies a correlation between Pt aggregation and the reduction of Pt to its metallic oxidation state. The Pt/Ani/Ar sample, annealed in argon at 200 °C, which is above the boiling point of aniline (184.1 °C), was observed to contain a large number of nanoparticles and only a few individual single atoms separately. This sample highlights the importance of aniline in the formation of Pt single atoms and clusters

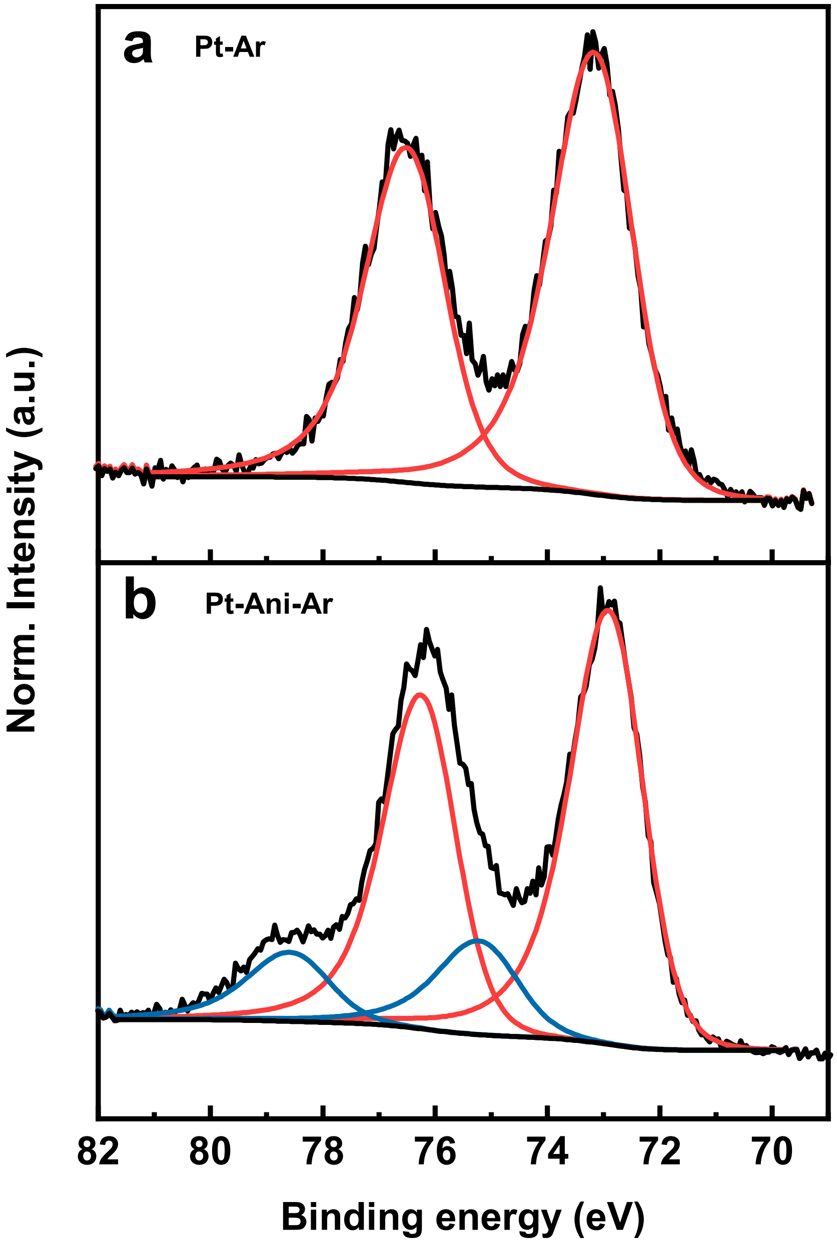

Figure S5. Repeated tests of Pt 4f XPS spectra of selected catalysts: (Pt/Ar) (**a**) and (Pt/Ani/Ar) (**b**). 20% of Pt(IV) component is also recorded in (Pt/Ani/Ar) sample.

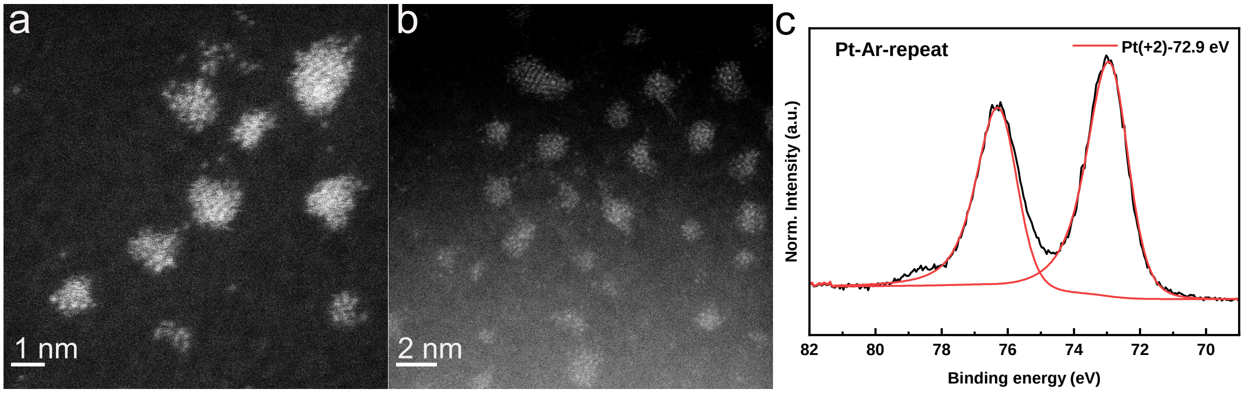

Figure S6. (**a,b**) STEM images of (Pt/Ar) sample showing the presence of Pt aggregates. (**c**) XPS spectra of a different (Pt/Ar) sample demonstrating the reproducibility of the XPS data in the manuscript.

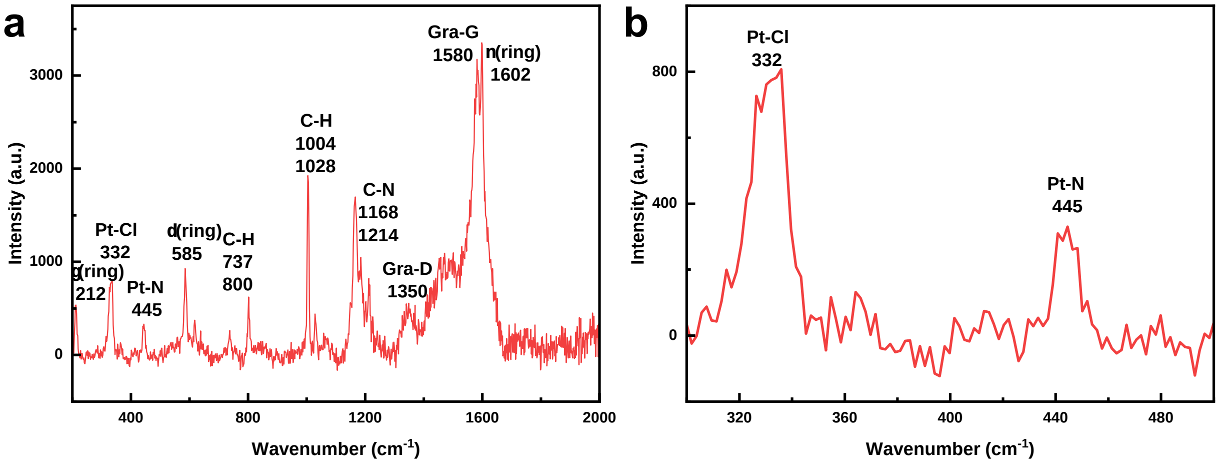

Figure S7. (**a**) Raman spectrum of the (Pt/Ani/Ar) sample.(**b**) The enlarged area from 300 cm^{-1} to 500 cm^{-1} in (**a**) highlights two characteristic peaks at 332 cm^{-1} (Pt-Cl) and 445 cm^{-1} (Pt-N).

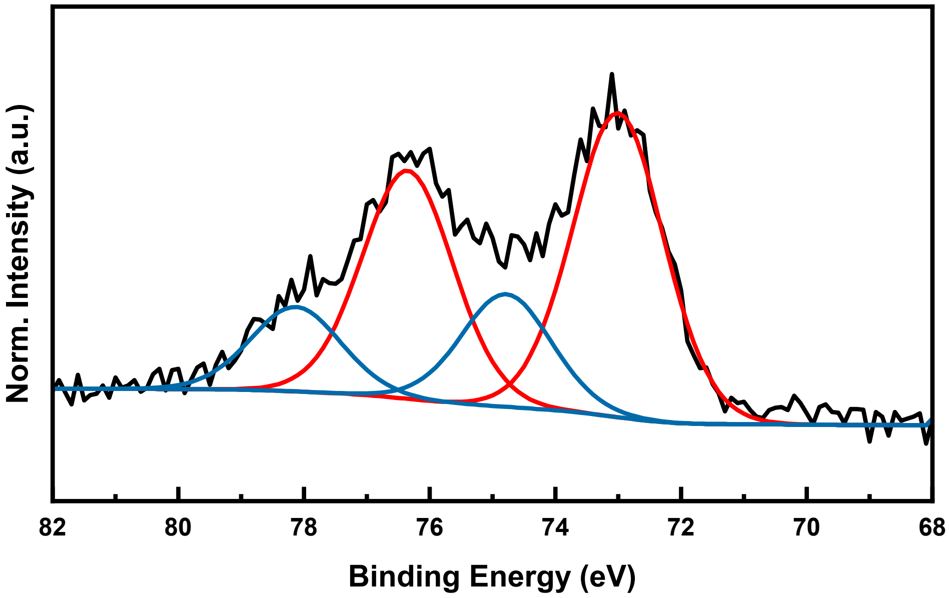

Figure S8. Fitted XPS spectra of Pt 4f for H_2PtCl_6 ethanol solution dispersed on GC, which has a 73.1% of Pt(II) and 26.9% of Pt(IV).

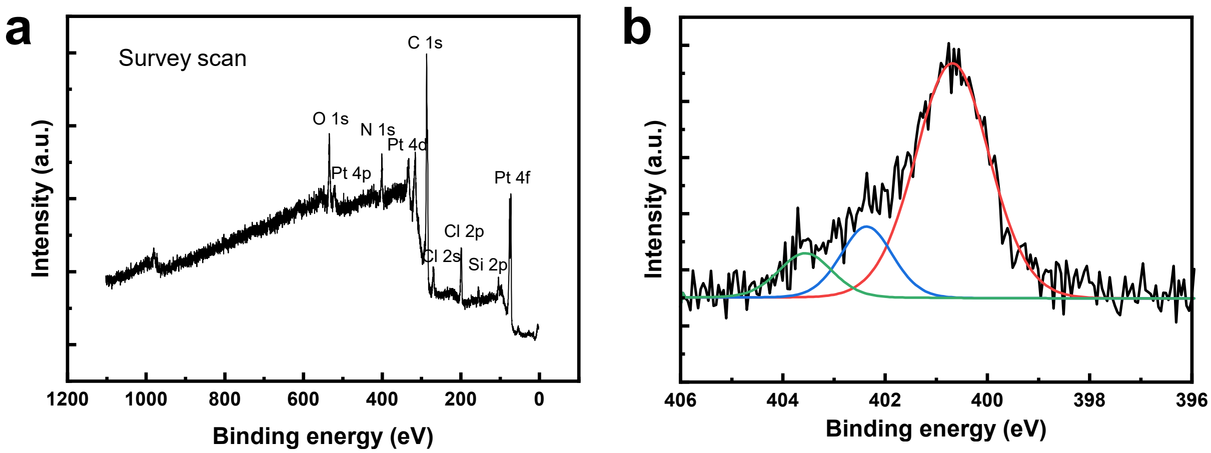

Figure S9. Survey XPS spectra (left) and high-resolution scan of N1s spectra (right) of (Pt/Ani/Ar).

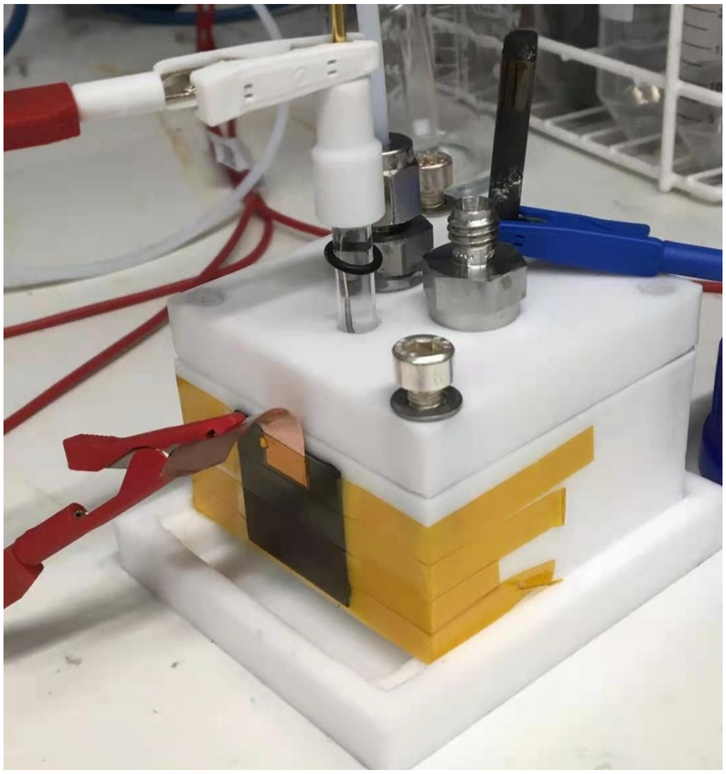

Figure S10. Setup of three-electrode cell employed for *operando* XAFS study at Diamond Light Source. A 1 mm thick glassy carbon with an area of 4 cm^2 was emplyed as a working conductive electrode to support the synthesised Pt single-site catalyst (same loading of 0.02-0.03 mg/cm^2 as lab-based setup). The GC with Pt catalysts was fixed to the window of the cell by PTFE tape. Then CA tests were conducted at different potentials. At each potential, we waited for the OCV to reach a stable electrochemical equilibrium condition and then applied a fixed potential. Once the current density fluctuation was within 0.01 mA/sec, XAFS data could be acquired.

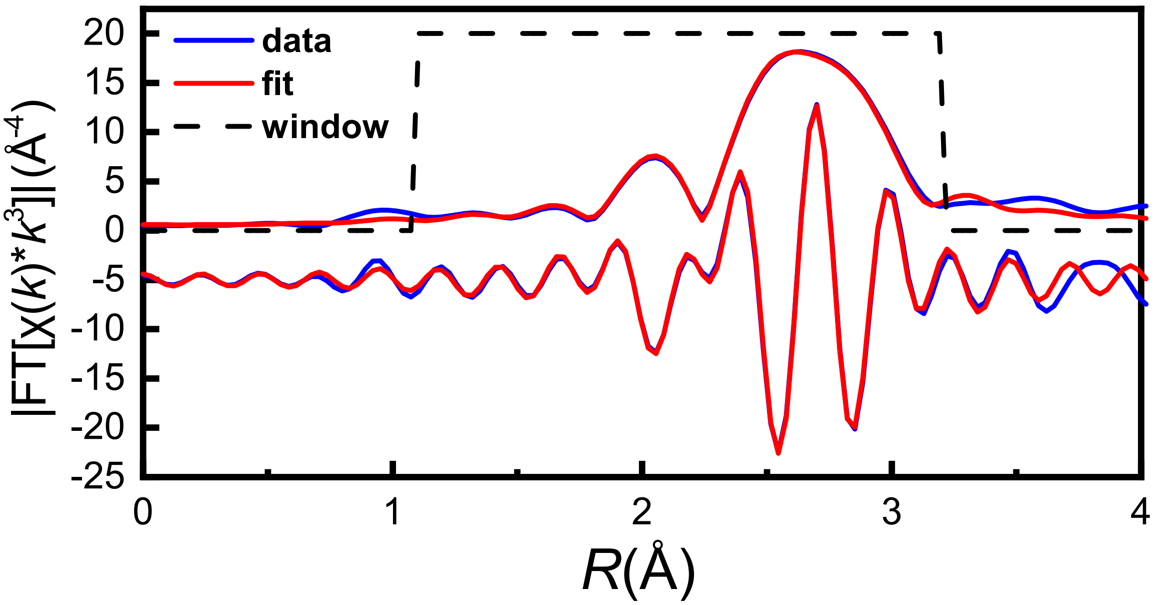

Figure S11. The experimental magnitude and real part component of the k^3-weighted Fourier transformed Pt L_3-edge EXAFS spectra (blue) of a Pt foil reference were fitted (red) in the range 1.15–3.2Å in R space. The experimental EXAFS data of Pt foil was fitted in R space (1.15-3.2 Å) (k-weighting=3) using a well-known model of bulk Pt (Space group Fm-3m, cell parameter a=0.3938 nm). The amplitude reduction factor was found to be 0.80 by setting the coordination number of Pt foil as 12 and guessing other parameters.

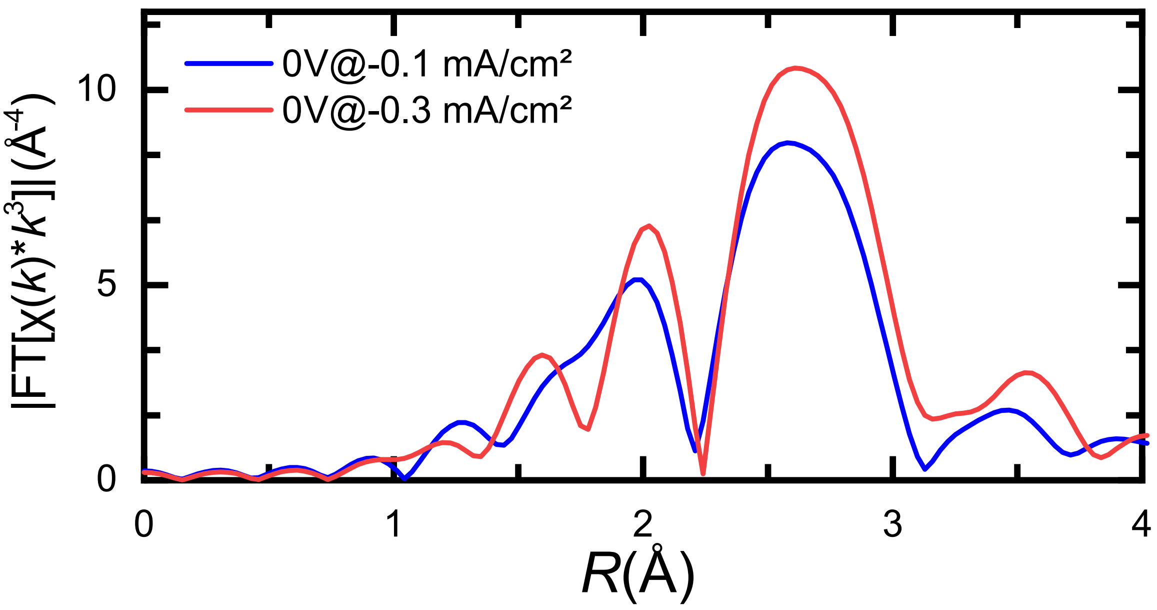

Figure S12. The corresponding magnitude component of the k^3-weighted Fourier Transformed Pt L_3-edge EXAFS spectra data before and after current jump at overpotential of 0 V

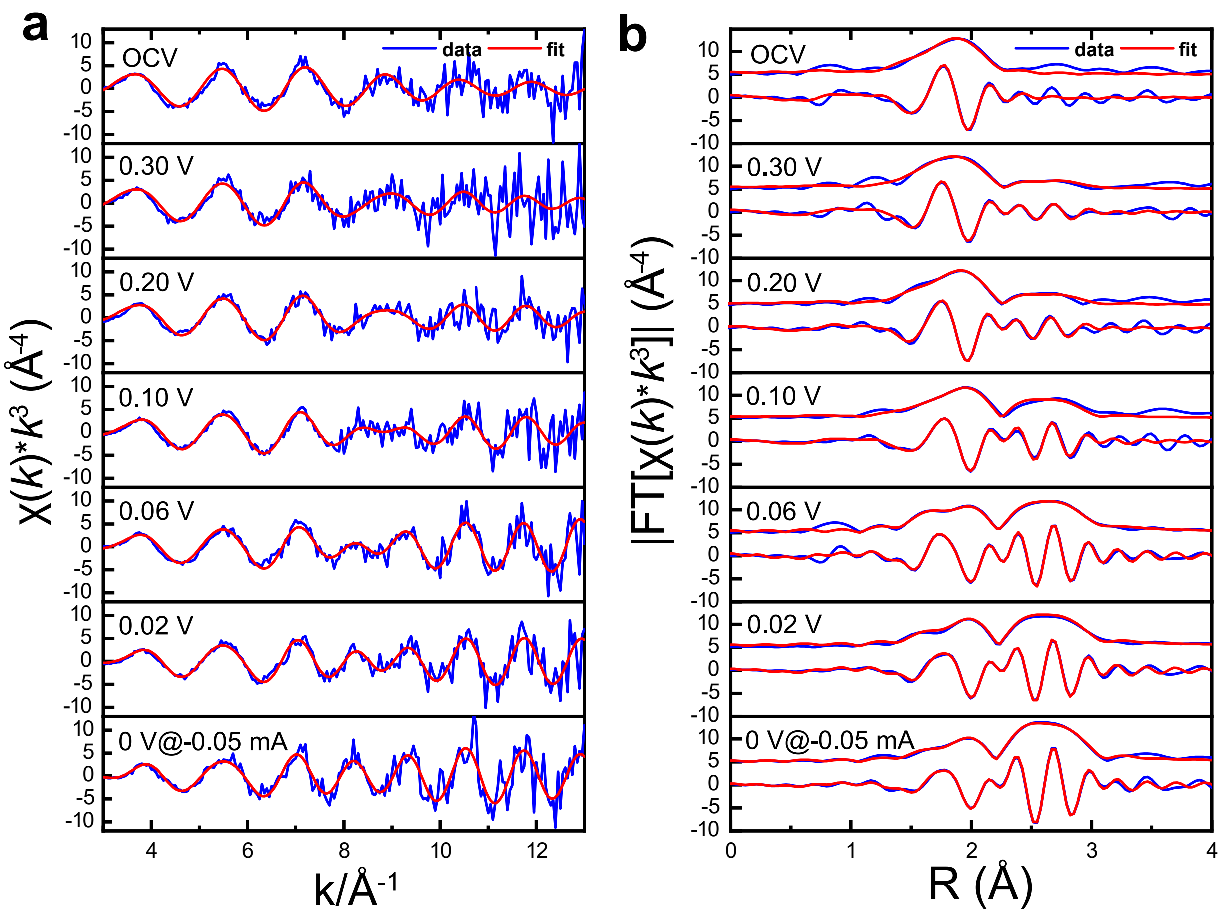

Figure S13. (**a**) Pt L_3-edge EXAFS spectra (blue) and corresponding curvefit (red) for Pt single-site complex at selected working potentials before 0 V, shown in k^3-weighted K-space (k range: 3 – 13 Å). (**b**) The experimental k^3-weighted Fourier-transformed EXAFS spectra data (blue) (magnitude and real part components) were fitted (red) in the range of 1.15 – 3.2 Å in R-space (OCV fitted in the range of 1.15-2.5 Å, and 0.30 V, 0.20 V fitted in the range of 1.15-3.0 Å) using calculated DFT model of $(C_6H_5NH_2)_2PtCl_2$ (Pt-N & Pt-Cl paths) and Pt foil (Pt-Pt path).

Table S1: Curvefit parameters for Pt L_3-edge EXAFS data in Operando experiment by DFT model of trans $(C_6H_5NH_2)_2PtCl_2$ and Pt foil. $S0^2$ is fixed as 0.80 and the fitted data range in K space and R space is: 3-12. 5Å, 1.1-2.5 Å for OCV; 3-13.5 Å, 1.15-3.0 Å for 0.10 V; 3-13 Å, 1.15-3.2 Å for other potentials. Debye Waller factors were constrained as δ^2(Pt-N)= δ^2(Pt-Cl) for reducing the number of variables. The numbers in brackets are uncertainties for each fitting result.

Sample	Path	CN	R(Å)	$\sigma(10^{-3}Å^2)$	R-factor
Pt foil	Pt-Pt	12	2.76	4.4(0.2)	0.002
OCV	Pt-N	2.4(0.7)	2.05	3.5(3.0)	0.008
	Pt-Cl	2.4(1.1)	2.30		
0.10 V	Pt-N	1.1(0.7)	2.00	6.1(3.3)	0.013
	Pt-Cl	2.8(1.1)	2.28		
	Pt-Pt	2.9(1.6)	2.74	5.3(2.6)	
0.06 V	Pt-N	1.0(0.5)	2.01	6.5(2.3)	0.004
	Pt-Cl	2.6(0.7)	2.26		
	Pt-Pt	3.1(0.6)	2.74	2.9(0.8)	
0.02 V	Pt-N	0.7(0.5)	2.00	6.6(2.7)	0.004
	Pt-Cl	2.2(0.7)	2.27		
	Pt-Pt	4.7(0.8)	2.74	4.6(0.9)	
0 V@0.05 mA	Pt-N	0.5(0.2)	1.97	2.9(2.0)	0.004
	Pt-Cl	1.2(0.3)	2.25		
	Pt-Pt	7.2(0.7)	2.75	5.6(0.5)	

Table S2: Curvefit parameters for Pt L_3-edge EXAFS data in operando experiment by DFT model of cis $(C_6H_5NH_2)_2PtCl_2$ and Pt foil. $S0^2$ is fixed as 0.80 and the fitted data range in K space and R space is: 3-12.5 Å, 1.1-2.5 Å for OCV; 3-13.5 Å, 1.15-3.0 Å for 0.10 V; 3-13 Å, 1.15-3.2 Å for other potentials. Debye Waller factors were constrained as δ^2(Pt-N)= δ^2(Pt-Cl) for reducing the number of variables. The numbers in brackets are uncertainties for each fitting result. The R-factor and uncertainties at some potentials are larger than the fitting results based on a trans structure.

<table>
<tr><th>Sample</th><th>Path</th><th>CN</th><th>R(Å)</th><th>σ(10^{-3}Å^2)</th><th>R-factor</th></tr>
<tr><td>Pt foil</td><td>Pt-Pt</td><td>12</td><td>2.76</td><td>4.4(0.2)</td><td>0.002</td></tr>
<tr><td rowspan="2">OCV</td><td>Pt-N</td><td>2.3(0.7)</td><td>2.05</td><td rowspan="2">3.4(2.9)</td><td rowspan="2">0.008</td></tr>
<tr><td>Pt-Cl</td><td>2.4(1.1)</td><td>2.29</td></tr>
<tr><td rowspan="3">0.10 V</td><td>Pt-N</td><td>0.9(0.7)</td><td>1.98</td><td rowspan="2">6.6(3.0)</td><td rowspan="3">0.018</td></tr>
<tr><td>Pt-Cl</td><td>3.1(1.1)</td><td>2.28</td></tr>
<tr><td>Pt-Pt</td><td>2.6(1.3)</td><td>2.74</td><td>4.8(2.2)</td></tr>
<tr><td rowspan="3">0.06 V</td><td>Pt-N</td><td>0.8(0.6)</td><td>2.01</td><td rowspan="2">6.6(2.3)</td><td rowspan="3">0.004</td></tr>
<tr><td>Pt-Cl</td><td>2.8(0.8)</td><td>2.26</td></tr>
<tr><td>Pt-Pt</td><td>3.1(0.6)</td><td>2.74</td><td>2.8(0.8)</td></tr>
<tr><td rowspan="3">0.02 V</td><td>Pt-N</td><td>0.8(0.3)</td><td>1.99</td><td rowspan="2">4.8(2.2)</td><td rowspan="3">0.005</td></tr>
<tr><td>Pt-Cl</td><td>1.8(0.5)</td><td>2.27</td></tr>
<tr><td>Pt-Pt</td><td>5.2(0.8)</td><td>2.74</td><td>5.0(0.7)</td></tr>
<tr><td rowspan="3">0 V@0.05 mA</td><td>Pt-N</td><td>0.5(2.4)</td><td>2.21</td><td rowspan="2">6.6(2.7)</td><td rowspan="3">0.007</td></tr>
<tr><td>Pt-Cl</td><td>2.0(1.9)</td><td>2.24</td></tr>
<tr><td>Pt-Pt</td><td>7.0(1.2)</td><td>2.74</td><td>5.6(0.8)</td></tr>
</table>

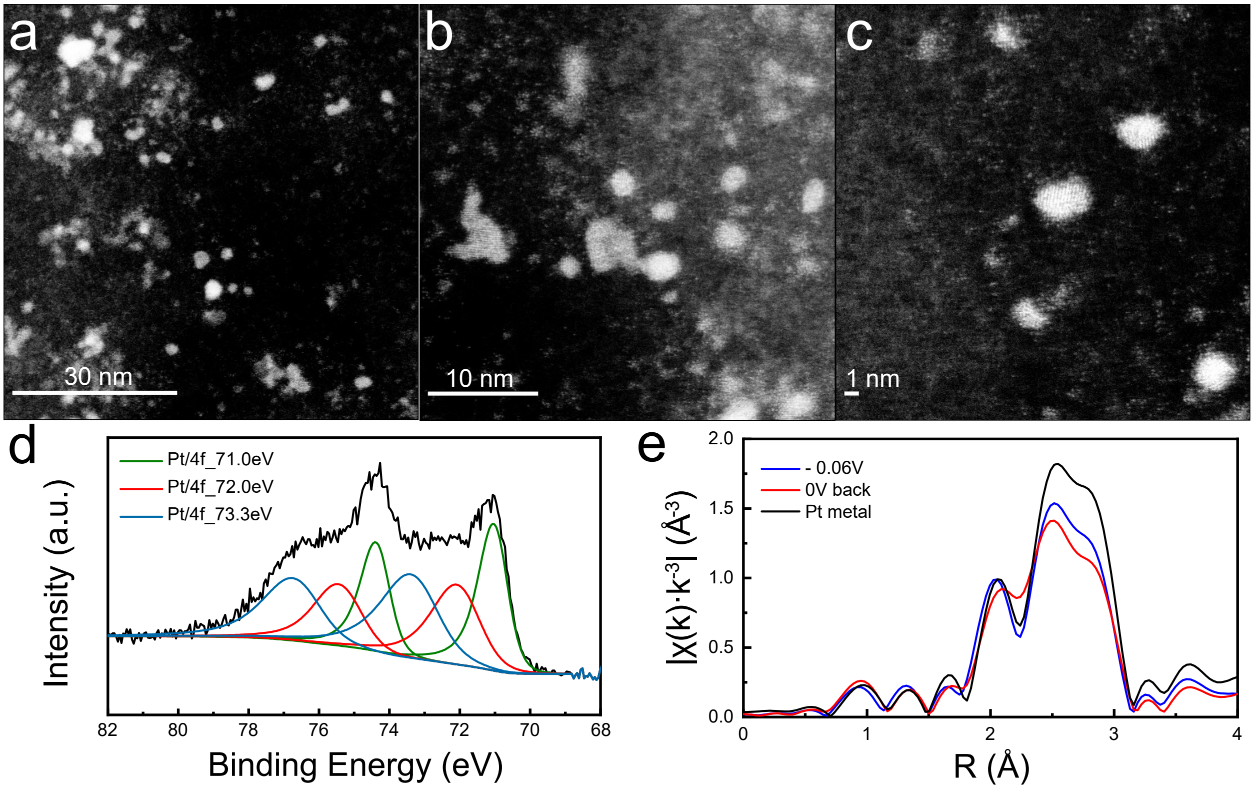

Figure S14. (**a**,**b**,**c**) STEM images of (Pt/Ani/Ar) sample post cycling show Pt nanoparticles. (**d**) Fitted XPS spectra of Pt 4f for a (Pt/Ani/Ar) sample post cycling which clearly shows the metallic Pt peaks (green peaks). (**e**) Pt L_3-edge EXAFS spectra of Pt foil and (Pt/Ani/Ar) sample at -0.06 V and 0 V vs RHE (return back from -0.06 V). The EXAFS spectra of metallic Pt peaks at 0 V shows that the formed Pt nanoparticles after cycling are irreversible.

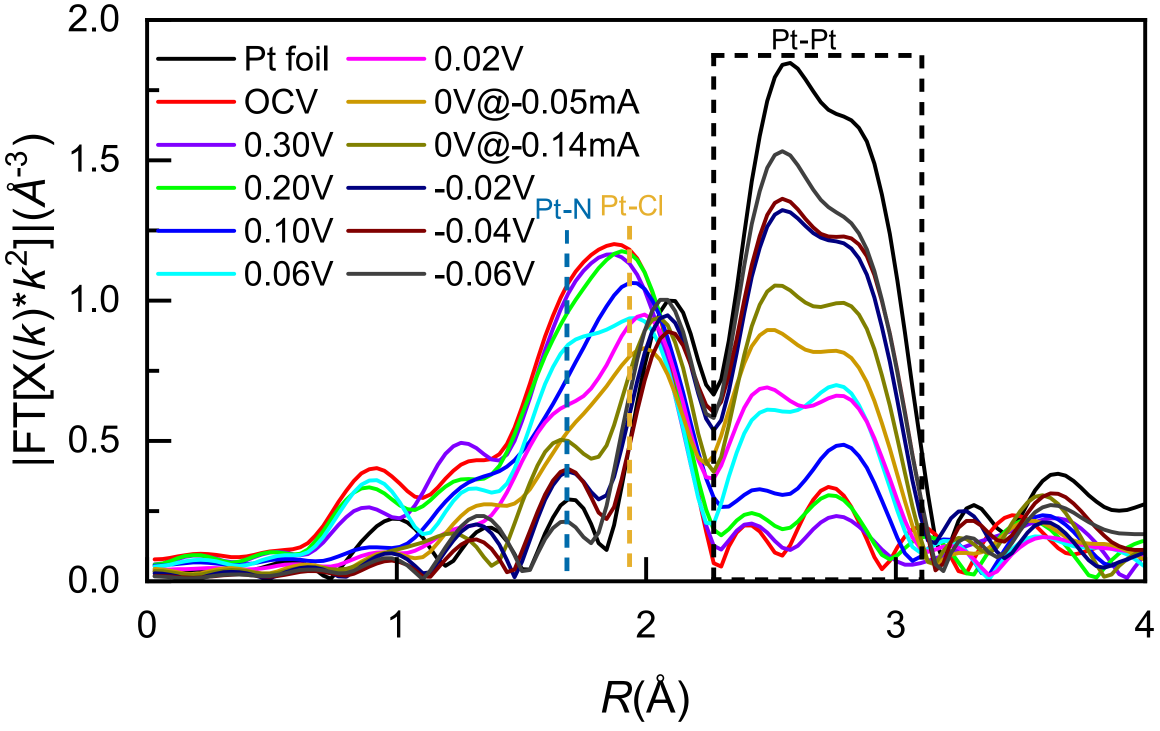

Figure S15. The corresponding K^2-weighted Fourier-transformed EXAFS spectra of (magnitude component) of (Pt/Ani/Ar) sample at different potentials during operando XAFS experiments. The vertical dashed lines or area show the Pt-N (blue), Pt-Cl (orange) and Pt-Pt (black) peaks.

References

(1) Ye, S.; Luo, F.; Zhang, Q.; Zhang, P.; Xu, T.; Wang, Q.; He, D.; Guo, L.; Zhang, Y.; He, C.; Others, Highly stable single Pt atomic sites anchored on aniline-stacked graphene for hydrogen evolution reaction. *Energy & Environmental Science* **2019**, *12*, 1000–1007.

(2) Fan, Y.; He, K.; Tan, H.; Speller, S.; Warner, J. H. Crack-free growth and transfer of continuous monolayer graphene grown on melted copper. *Chemistry of Materials* **2014**, *26*, 4984–4991.

(3) Ravel, B.; Newville, M. ATHENA, ARTEMIS, HEPHAESTUS: data analysis for X-ray absorption spectroscopy using IFEFFIT. *Journal of synchrotron radiation* **2005**, *12*, 537–541.

(4) Kresse, G.; Furthmüller, J.; Hafner, J. Theory of the crystal structures of selenium and tellurium: the effect of generalized-gradient corrections to the local-density approximation. *Physical Review B* **1994**, *50*, 13181.

(5) Kresse, G.; Furthmüller, J. Efficiency of ab-initio total energy calculations for metals and semiconductors using a plane-wave basis set. *Computational Materials Science* **1996**, *6*, 15–50.
